# The Limited Predictive Power of the Pauling Rules†

**DOI:** 10.1002/anie.202000829

**Published:** 2020-03-20

**Authors:** Janine George, David Waroquiers, Davide Di Stefano, Guido Petretto, Gian‐Marco Rignanese, Geoffroy Hautier

**Affiliations:** ^1^ Institute of Condensed Matter and Nanosciences Université catholique de Louvain Chemin des étoiles 8 1348 Louvain-la-Neuve Belgium

**Keywords:** computational chemistry, coordination environments, materials informatics, solid-state structures

## Abstract

The Pauling rules have been used for decades to rationalise the crystal structures of ionic compounds. Despite their importance, there has been no statistical assessment of the performances of these five empirical rules so far. Here, we rigorously and automatically test all five Pauling rules for a large data set of around 5000 known oxides. We discuss each Pauling rule separately, stressing their limits and range of application in terms of chemistries and structures. We conclude that only 13 % of the oxides simultaneously satisfy the last four rules, indicating a much lower predictive power than expected.

## Graphical abstract

**Exceptions to the rules**: The statistical analysis of the five Pauling rules reveals their limited predictive power. Each of the Pauling rules is tested on a set of 5000 experimentally known oxides. Their limits in terms of chemistries and structures are discussed in detail. Only 13 % of all tested structures fulfil the last four rules simultaneously.

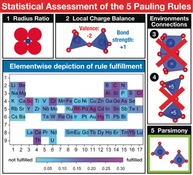

## Introduction

Understanding and predicting the crystal structure of inorganic materials is an important goal of chemistry. In 1929, Linus Pauling published a series of five empirical rules rationalising inorganic crystal structures.^1^ The Pauling rules apply to ionic compounds and describe what are the preferred local environments of a cation and how these environments connect to each other. These rules have become a cornerstone of solid‐state chemistry and remain the main empirical theory rationalising crystal‐structure stability. Pauling developed these five rules by combining his knowledge of inorganic crystal structures and simple electrostatic arguments. Though, in response to observed deviations, these rules have been slightly improved over the years, nowadays they remain widely used in their original form.^2^, ^3^, ^4^, ^5^, ^6^, ^7^, ^8^

The Pauling rules are not laws of nature. It is thus expected that they are not always correct. While previous studies have looked at their application on specific chemistries (for example, silicates) or at their fundamental orbital origin,^9^, ^10^, ^11^, ^12^, ^13^ they have not yet been assessed statistically on a large scale. The absence of such a rigorous assessment of the validity of the Pauling rules inhibits their use for true prediction and prevents the development of improved and alternative rules. Building on recent advances in crystal‐structure‐analysis tools including the automatic identification of local environments and their connectivity,^14^, ^15^, ^16^ we report here on the first statistical evaluation of the Pauling rules on several thousands of compounds. Our work shines light on their strengths and limits, enables a more cautious use of them and offers a first necessary step towards their future improvement.

## Results and Discussion

Our analysis relies on the use of a tool for automatic local‐environment detection on a set of oxides coming from the Inorganic Crystal Structure Database (ICSD) and present in the Materials Project database (see Supporting Information for a detailed description).^17^ We focused on oxides as they are ionic enough for the Pauling rules to be applicable and because the large number of oxides known makes it possible to obtain a large data set and hence good statistics. In total, more than 5000 oxides (a subset of the structures from Ref. 14) are considered. The paper presents and discusses the performance of each of the five rules individually and wraps up by commenting on the overall quality of the five rules taken altogether.

### Rule 1: Radius‐Ratio Rule

**Figure 1:**
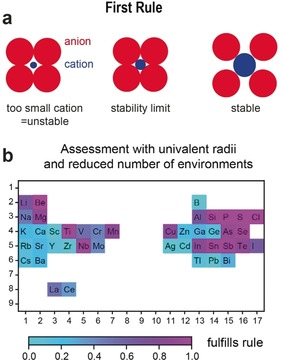
Assessment of the first rule. a) Illustration of the radius‐ratio rule (Pauling's first rule). The figure was inspired by Ref. 18. b) Percentages of correct predictions by Pauling's rule to all tested predictions. The prediction is correct if it agrees with the geometrical assessment of the structure.

The first rule states that “[…] The coordination number of the cation [is determined] by the radius ratio [of cation and anion].”^1^ This rule is based on a hard‐sphere model of the atoms as shown in Figure 1 a. A coordination environment is stable only if the radius ratio of cation and anion falls within the geometrically derived stability window of this environment.

While several atomic‐ and ionic‐radii schemes were developed after Pauling's original work, we used the simplest here—Pauling's univalent radii.^7^ We found a rather unsatisfactory agreement with the first rule in our data set. Only 66 % of the tested local environments agreed with the expectation from Pauling's first rule. Figure 1 b shows an analysis of the fulfilment of the rule by element. By design, the first rule can only work for elements presenting a low diversity in local environments (for instance, Si, P, and S are mainly tetrahedral).^14^ In contrast, many of the alkali and alkaline‐earth metals and some transition metals are found in a variety of environments and present strong deviations from the rule. We link the failure of the first rule to an inadequate fundamental assumption that a given cation should always be found in one and only one type of local environment. Crystal chemistry is more complex and many cations show diversity in their possible local environments in oxides (see Figure S1 in the Supporting Information).^14^ While other ionic radii have been proposed to improve the first rule, we note that some of them such as the Shannon radii^6^ use ionic radii depending on the local environment and cannot be used for local‐environment prediction. The limits of the first rule were already pointed out by Burdett based on a smaller data set.^10^

### Rule 2: Electrostatic‐Valence Rule

**Figure 2:**
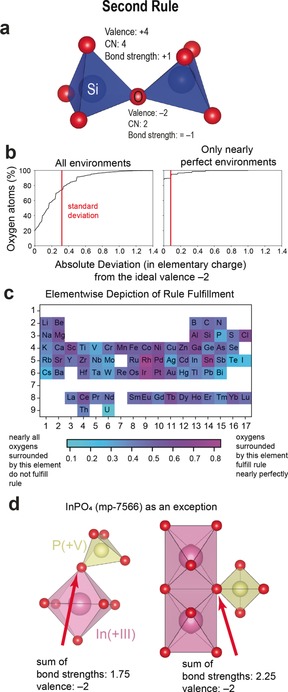
Assessment of the second rule. a) Illustration of the electrostatic‐valence rule. b) Share of the oxygen atoms that show, at most, a certain absolute deviation from the ideal valence −2 for all environments (left) and for structures with only very symmetric coordination environments (right). c) Elementwise depiction of the fulfilment of the second rule. d) Depiction of relevant connections of polyhedra for the calculation of the sum of bond strengths within the crystal structure of InPO_4_. The sum of bond strengths arrives, in one case, at 2.25 and, in the other, at 1.75.

The second rule focuses on local charge compensation within crystal structures: “In a stable coordination structure the electric charge of each anion tends to compensate the strength of the electrostatic valence bonds reaching to it from the cations at the centres of the polyhedra of which it forms a corner […].”^1^ The application of this rule is demonstrated on α‐quartz (see Figure 2 a). The charge of the oxygen atom (−2) is compensated by the bond strengths of the two silicon neighbours (+1). The bond strength of the bond from the cation is calculated by dividing the valence of the cation by its coordination number (+4/4).

We tested the second rule for all oxygen atoms in our data set (Figure 2 b, left). The rule is only nearly exactly fulfilled for roughly 20 % of all oxygen atoms (an absolute deviation of 0.01 is allowed). Extreme deviations from the second rule are observed in some common structures such as pyrochlores. When mixing 2+ and 5+ cations (for example, Cd_2_Ta_2_O_7_, mp‐5548),^19^ the O in the 2*a* Wyckoff position in pyrochlore shows a sum of bond strengths of one (vs. an expected value of 2).

Figure 2 c indicates the fulfilment of the second rule per element. There is no clear trend within the periodic table. Oxygen atoms surrounded by Al, Si, and Sn as well as certain transition metals (Sc, Rh, Pd, Ir) satisfy this rule best (≈60 %). In contrast, oxygen atoms surrounded by P deviate strongly from the rule despite its proximity to Si in the periodic table. Many phosphates and vanadates are, in fact, among the exceptions to the second rule, for example, InPO_4_ (Materials Project ID: mp‐7566)^20^ and CrVO_4_ 21 (mp‐19418), which crystallise in the same structure type. InPO_4_ and the calculation of the bond‐strength sums are depicted in Figure 2 d. In this simple structure, the oxygen atoms show a sum of bond strengths deviating by 12.5 % from the nominal +2 value. Deviations to the second rule had already been identified by several authors in the past and extensions had been proposed.^2^, ^4^, ^11^, ^12^, ^22^, ^23^, ^24^

According to Baur,^2^ distortions of chemical environments could accommodate deviations from local charge compensation. Our data agrees with this hypothesis and a subset of highly symmetrical, undistorted local environments (221 materials instead of ≈5100) leads to a nearly perfect fulfilment of the rule as shown in Figure 2 b (right). In a nutshell, the second rule can be powerful, but only for very symmetric structures with undistorted environments. Unfortunately, these perfect structures are a minority of the existing oxides.

### Rule 3: The Sharing of Edges and Faces

**Figure 3:**
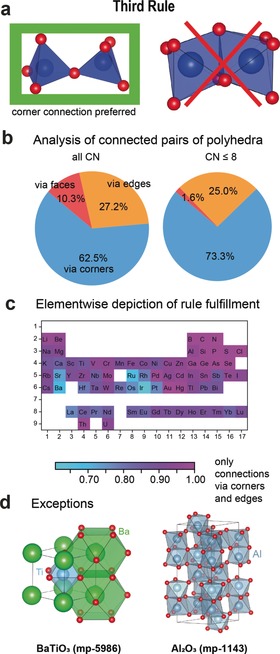
Assessment of the third rule. a) Illustration of the rule on “the sharing of edges and faces”. In stable crystals, corner connections of coordination polyhedra are preferred over edge and especially face connections. b) Shares of connected pairs of polyhedra that are connected via corners, edges, and faces for all coordination polyhedra (left) and only coordination polyhedra with a coordination number (CN) equal to 8 or smaller (right). c) Elementwise fulfilment of the rule. d) Structure of BaTiO_3_ (mp‐5986)^27^, ^28^ and Al_2_O_3_ (corundum, mp‐1143) showing connections of polyhedra via faces.

The third rule links the stability of crystal structures with the type of connections between the coordination polyhedra, as illustrated in Figure 3. “The presence of shared edges, and particularly of shared faces, in a coordinated structure decreases its stability; […].”^1^ Simple electrostatic arguments can justify such a rule as cations will be closer when sharing edges and faces.

We tested the third rule by identifying all connected pairs of polyhedra and computing the fraction of the connected pairs that are corner‐ (63 %), edge‐ (27 %), or face‐sharing (10 %; see Figure 3 b, left). This agrees well with Pauling's rule. To probe the effect of chemistry on the third rule, we show in Figure 3 c how often face‐sharing connections are present for different cations. In general, smaller elements seem to fulfil the rule better (low period and high group number). This is confirmed by a clear dependence of cation connectivity with atomic radius (see Figure S8 in the Supporting Information). Many of the “deviations” to the rule, like the presence of face‐sharing polyhedra, come from larger cations. For instance, the very common perovskite structure shows face‐sharing polyhedra but only for the large‐cation A site (for example, Ba^2+^ in BaTiO_3_).

In fact, if we exclude cations with high coordination numbers from our analysis (considering only coordination numbers ≤8), we obtain an even better agreement with Pauling's third rule with less than two percent of face‐sharing environments (see Figure 3 b, right). Structures with face‐sharing low‐coordination ions such as the corundum structure (Al_2_O_3_, mp‐1143, see Figure 3 d)^25^, ^26^ are quite exceptional. The importance of the coordination number is rationalised by its link to ionic size and to cation–cation distances. Larger ions will have larger cation–cation distances and lower electrostatics.

### Rule 4: The Nature of Contiguous Polyhedra

**Figure 4:**
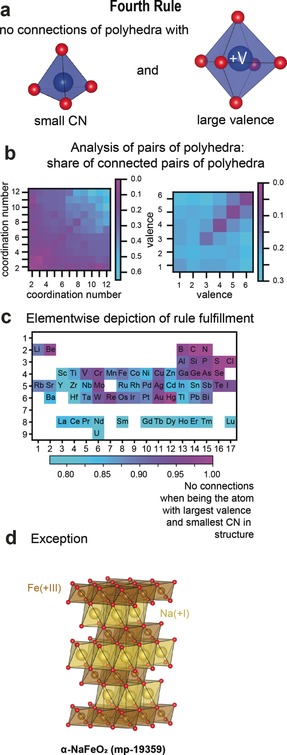
Assessment of the fourth rule. a) Illustration of the fourth rule which deals with “the nature of contiguous polyhedra”. b) The dependency of the share of connected pairs of polyhedra of all considered pairs of polyhedra on the coordination numbers, and valences of the cations. c) Elementwise depiction of the rule fulfilment. d) One of the exceptions, α‐NaFeO_2_, is shown. The octahedral coordination environments of Fe^+III^ show connections. Na^+I^ is also octahedrally coordinated so that Fe^+III^ is the cation with the lowest coordination number and the highest valence.

The fourth rule is an extension of the third rule. It focuses on crystals containing different cations and indicates how they should connect depending on their oxidation state and local environment (see Figure 4 a): “In a crystal containing different cations those with large valence and small coordination number tend not to share polyhedron elements with each other.”^1^

Similar to the third rule, we analysed pairs of polyhedra but included non‐connected pairs of polyhedra as well. We plotted the share of connected pairs of all considered pairs of polyhedra (selected by a distance criterion) as a function of the coordination numbers and as a function of the valences (see Figure 4 b). We find that the coordination number strongly affects the tendency for two polyhedra to be connected. Cations with lower coordination numbers tend to clearly be less connected to each other. This is in good agreement with Pauling's fourth rule. However, the oxidation state surprisingly does not seem to affect the connectivity between polyhedra, in clear disagreement with the fourth rule. The exceptions to the fourth rule (that is, structures in which the polyhedra of cations with the highest valence and smallest coordination number are connected) amount to 40 % of all tested structures. Many structures mixing octahedral sites filled with a high‐valent cation and higher‐coordination‐number sites with low‐valent cations do not fulfil the fourth rule. This is the case, for instance, for the perovskite (that is, BaTiO_3_ (mp‐5986)),^27^, ^28^ La_3_Nb_2_O_7_ (mp‐560349),^29^ and pyrocholore structures (for example, Nb_2_Cd_2_O_7_ (mp‐5472)).^30^ In all these examples, the high‐valent (4+ or 5+) octahedral environments are directly connected together, in direct violation of the fourth rule. This indicates that covalent and electronic‐structure effects might be more important than electrostatics in these compounds. Additionally, structures belonging to the α‐NaFeO_2_ structure type^31^ (mp‐19359, see Figure 4 d for a depiction of the structure) are also among the exceptions. Here, all cations are octahedrally coordinated and the cations with the largest valence share polyhedron elements with each other. In the specific case of α‐NaFeO_2_, this deviation from the Pauling rules might be rationalised with the help of antiferromagnetic interactions of the iron atoms,^32^, ^33^ which might stabilise the structure only when the Fe ions are direct neighbours.

Figure 4 c shows the dependence of the fourth rule on chemistry. Main‐group elements in general follow the rule more than other chemistries such as transition metals. Within the main‐group elements, we see, however, strong differences. For instance, sulfur (in SO_4_^2−^ groups) almost always fulfils the rule, while this is less the case for phosphorus. This comes from the less common condensation of polyanions in sulfates compared to phosphates with, for instance, 128 structures forming P_2_O_7_^4−^ groups while S_2_O_7_^2−^ groups are rare (5 within our database).

### Rule 5: The Rule of Parsimony

**Figure 5:**
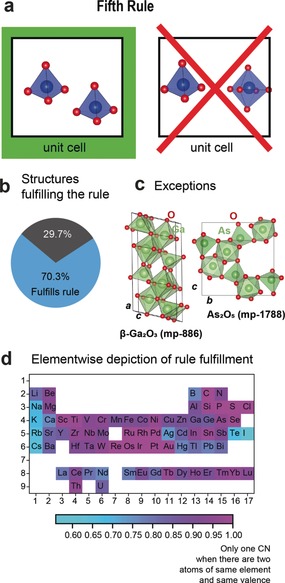
Assessment of the fifth rule. a) Visualisation of the rule of parsimony. b) Pie chart depicting the structures fulfilling the rule of parsimony (only coordination numbers are considered). c) Exceptions: crystal structures of β‐Ga_2_O_3_ 34, 35 and As_2_O_5_.^36^ Both β‐Ga_2_O_3_ and As_2_O_5_ 34, 35 show tetrahedral and octahedral coordination of their cations in the same structure. d) Elementwise fulfilment of the fifth rule when only coordination numbers are considered.

The fifth rule states the number of constituents in stable crystal structures. According to Pauling, “The number of essentially different kinds of constituents in a crystal tends to be small.”^1^ In other words, a cation (an element in a given positive oxidation state) prefers to occupy the same local environment in a given crystal structure. For instance, it would be unfavourable for a cation to be tetrahedral and octahedral in the same compound (see Figure 5 a).

Around 70 % of all tested structures show the same local environment for their cation types (see Figure 5 b). The elementwise analysis of the rule fulfilment is depicted in Figure 5 d. Alkali and alkaline‐earth metals deviate from this rule and are easily present in different local environments in the same structure. The wide range of possible local environment for alkali and alkaline‐earth metals makes the breaking of the fifth rule not surprising. More surprisingly, a few main‐group elements such as B, Ga, or Ge also break the parsimony rule quite commonly. Borates easily mix tetrahedral and trigonal‐planar borate groups such as in CaB_2_O_4_ (mp‐8056).^37^ Exceptions to the rule are even found in simple binaries: β‐Ga_2_O_3_ (mp‐886)^34^, ^35^ and As_2_O_5_ (mp‐1788) contain Ga(+III) or As(+V) in fourfold and sixfold coordination within the same compound (see Figure 5 c).^36^

### Combined Assessment of the Five Rules

To assess the overall performance of the Pauling rules, we combine the assessment of the second to fifth rules. We avoid assessing the first rule, because it will strongly depend on the quality of the univalent radii. Additionally, we do not believe that any simple rule directly linking ionic radius to local environment will ever be predictive in view of the local‐environment diversity of many cations.

**Figure 6:**
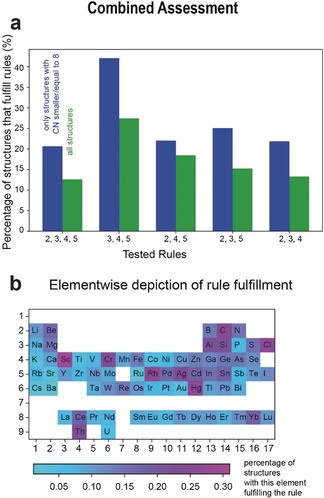
Combined assessment of the second to fifth rules. a) Dependency of the rule fulfilment on each of the rules and the influence of the coordination numbers (CN) on the rule fulfilment. b) Percentages of structures including this element which fulfil the rule.

We found a few structure prototypes that fulfil the four Pauling rules: the rutile structure type, for example, SnO_2_ (mp‐856), spinels such as MgAl_2_O_4_ 38, 39 (mp‐3536), structures in the scheelite structure type (CaWO_4_) such as BiAsO_4_ 40 (mp‐561068), and many structures in the ZrSiO_4_ (mp‐4820) structure type. Furthermore, some phosphates such as α‐AlPO_4_ (mp‐3955, berlinite), which is isotypic to α‐quartz,^41^, ^42^ several sulfates such as In_2_(SO_4_)_3_ (mp‐541450),^43^ and silicates such as Mg_2_SiO_4_ (mp‐2895)^44^ are among the fulfilling structures. If one looks at the elementwise fulfilment of these four rules in Figure 6 b, one can see that structures including Al and Si tend to fulfil the rule better than structures including P and the alkali and alkaline‐earth metals.

Overall, only 13 % of all tested structures fulfil the second to fifth rules simultaneously. This indicates that structures with at least one deviation to the Pauling rules are frequent, and it demonstrates that the predictive power of the Pauling rules is quite limited. To find out which rules are the most problematic, we show in Figure 6 a how our conclusion varies if some rules are disregarded. Each green bar indicates the success rate of the Pauling rules if the four rules are combined or only a subset of three rules is applied. The second rule leads to the most exceptions and has the largest influence on the overall fulfilment of the rules. As the previous analysis of the rules indicated the different behaviour of high‐ vs. low‐coordination environments, we plot in Figure 6 a (in blue) the fulfilment of the rule when excluding cations in high‐coordination environments. Even in those more favourable conditions, the improvement is modest, with 20 % of compounds fulfilling the four rules.

## Conclusion

We presented the first statistical analysis of all five Pauling rules, focusing on their individual performances and their dependency on chemistry. In our opinion, the first rule, while commonly used, is the most problematic. Indeed, there are many cations that show a large diversity of local environments and fundamentally cannot be correctly described by the first rule. Instead of using the radius‐ratio rule, we suggest using statistics of the coordination environments to check if the coordination environment found is usual or not.

As for the other rules, only 13 % of all tested structures fulfil the second to fifth rules simultaneously. Our results cast doubt on the real predictive power of the Pauling rules. The success rate of the rules can be improved by narrowing their application to certain regions of the periodic table or to certain cations. Restricting the rules to certain chemical families does not appear very promising, as no clear chemical trend across all rules is observed and elements as chemically similar as Si and P obey the rules very differently. Ionic size appears more important and removing large coordination environments (>8) from the analysis improves the third and fourth rules significantly. Likewise, only considering undistorted, very symmetric local environments improves the second rule drastically. Our findings about the true scope of different Pauling rules will be very helpful to the solid‐state chemists commonly applying them. However, restricting the rules to specific cases lowers the universal ambition of the original Pauling rules, as the vast majority of oxides lie outside these restrictions. Our work therefore calls for the development of new empirical rules beyond the almost one‐century‐old Pauling rules. Our analysis and the data set of connectivity and local environment provided is a first step towards building this new theory that could potentially benefit from the recent growth in the use of machine‐learning techniques in chemistry and materials science.^45^, ^46^

## Conflict of interest

The authors declare no conflict of interest.

## Supporting information

As a service to our authors and readers, this journal provides supporting information supplied by the authors. Such materials are peer reviewed and may be re‐organized for online delivery, but are not copy‐edited or typeset. Technical support issues arising from supporting information (other than missing files) should be addressed to the authors.

Supplementary
