# Supplementary material for: The Limited Predictive Power of the Pauling Rules
Source: Angew Chem Int Ed Engl. 2020 Mar 20;59(19):7569–75. doi: 10.1002/anie.202000829 (PMC7217010; doi:10.1002/anie.202000829)
Supplement: Supplementary file 1 — Supplementary [file ANIE-59-7569-s001.pdf]

Angewandte Chemie
Eine Zeitschrift der Gesellschaft Deutscher Chemiker

Supporting Information

The Limited Predictive Power of the Pauling Rules**

*Janine George, David Waroquiers, Davide Di Stefano, Guido Petretto, Gian-Marco Rignanese, and Geoffroy Hautier**

anie_202000829_sm_miscellaneous_information.pdf

Supporting Information

Experimental Section

We analysed the *ab initio* relaxed structures of around 5100 oxides from the Materials Project Database that originally stem from the experimental part of the Inorganic Crystal Structure Database (ICSD)[1] to ensure that no pathologically unstable structures are included in our analysis. We only focused on materials with an energy above hull smaller or equal to 0.025 eV/atom (status on 10.4.2019). This ab initio relaxation corrects for uncertainties of the bond lengths and especially the coordination environments within the original structure predictions via X-ray or neutron diffraction. We arrive at quantitatively similar results if we use purely experimental data of 5000 oxides from the Crystallography Open Database (Figures S2-S7 in this Supporting Information).[2-6] We ensured that no duplicates were present in this data set and we worked with the primitive cells. The oxidation states are determined with the help of a bond-valence analysis that is implemented in pymatgen.[7] If this analysis failed, we used the oxidation states as given in the ICSD (only for the Materials Project data).[8] To analyse the coordination environments, we used the ChemEnv package with the default MultiWeigthsChemEnvStrategy, that was developed in the previous study on the statistics of coordination environments in oxides.[8] We applied it to a primitive unit cell of the oxide. To analyse the Pauling rules, we then only used the coordination environment that was assigned with the highest confidence (i.e. highest "ce_fraction"). The oxides from the Materials Project have already been studied in reference [8] and the assignment of the oxidation states was based on this study.
The code to analyse each of the Pauling rules can be accessed via https://github.com/jageo/paulingpublication (v1.01; an archived version can be found here: https://doi.org/10.5281/zenodo.3654428) and it is described in detail below.
CSV Files including the structures fulfilling and not fulfilling the second to fifth rules are included as a Supporting Information and are additionally archived on https://doi.org/10.5281/zenodo.3654989.
All crystal structures have been depicted with VESTA.[9]

Description of the code to analyse the Pauling rules:

To test the first rule, we compared the coordination environments predicted by the Pauling rule with the coordination environments as evaluated by the ChemEnv package. We only considered those environments for which the algorithm implemented in the ChemEnv packages resulted in one of the 7 environments that Pauling first used and that are listed in Table S1 (~ 40404 environments). To calculate the radius ratio, we used the univalent radii that Pauling developed.[10] Those radii only exist for a limited number of cations and they have the advantage that they do not depend on the coordination number and the valences.
To assess how well the first rule can work in principle, we calculated the Shannon entropy of the environments for each element and divided it by the maximal possible Shannon entropy (there are 66 different environments implemented).

Table S1. Environments and corresponding radius ratio windows that were considered in our analysis of the radius ratio rule.

Environment	Radius Ratio Window
Tetrahedron	≥0.225, <0.414
Octahedron	≥0.414, <0.592
Face-Capped Octahedron	≥0.592, <0.645
Square Antiprism	≥0.645, <0.732
Tricapped Triangular Prism or Cube	≥0.732, <1.0
Cuboctahedron	≥1.0

To assess the second rule, we calculated bond valence sums for each oxygen atom present in our data set and compared them to the ideal valence of oxygen in an oxide (-2). To focus on only very symmetric environments, we used environments with a continuous symmetry measure smaller than 0.1 and a coordination environment fraction higher than 0.95. To arrive at an elementwise analysis, we analysed the ratio of the number of cations around the oxygens that fulfil the rule to the number of all cations of around investigated oxygens for each element.

To analyse the third rule, we focused on connected pairs of polyhedra within the stable oxides, which show a cation-cation distance smaller or equal to 8 Å, and analysed their connections. We defined exceptional structures as only those that show face connections. To arrive at an elementwise analysis of the exceptions, we calculated the ratio of the number of cations that showed corner and edge connections to the number of all cations that showed connections for each element.

To analyse the fourth rule, we again focused on pairs of polyhedra, which show a cation-cation distance smaller or equal to 8 Å, within stable oxides that have different cations (i.e. they differ in valence or coordination number, ~ 4938 compounds), and analysed their connections as a function of the coordination numbers or the valences in the polyhedra pairs. We defined exceptions to this rule as structures where those cations with the smallest coordination number and largest valence that is present in the structure, show connected polyhedra. The latter version is the least strict interpretation of the fourth rule. We stress that other definitions of the exceptions would have been possible. To arrive at an elementwise analysis of the exceptions, we calculated the ratio of the number of cations with the highest valence and smallest coordination number that showed no connections to the number of cations with the highest valence and smallest coordination number for each element.

To analyse the fifth rule, we only looked at crystals having at least two chemical identical cations (i.e. the same element with the same valence, 5006 compounds) within the primitive unit cell. We then tested whether chemical identical cations showed the same coordination number. For the elementwise analysis, we calculated the ratio of the number of chemical identical cations that showed the same coordination number and the number of all chemical identical cations for each element.

To analyse rules two to five, we furthermore assumed that the fourth and fifth rule are fulfilled if the precondition for testing these rules is not fulfilled (i.e. they don’t have cations differing in their valences or coordination numbers, or they don’t have more than one cation of the same

element and valence in the primitive unit cell). This ensured that we could also test compounds such as α-SiO_2 in the overall analysis (5114 compounds were analysed).
To guarantee a significant result, we only showed elements in the elementwise depiction of the fulfillment of the rules for which more than 50 coordination environments (rules 1-5) or 25 structures including this element (combined assessment of rule 2-5) were present. The number of environments that were considered in the study are shown in the Figure S9. It demonstrates that conclusions drawn for P containing compounds are very reliable.
In the case of the experimental structures from the Crystallography Open Database (COD), we used 50 structures as a limit for the elementwise analysis of the combined assessment of the rules 2 to 5.

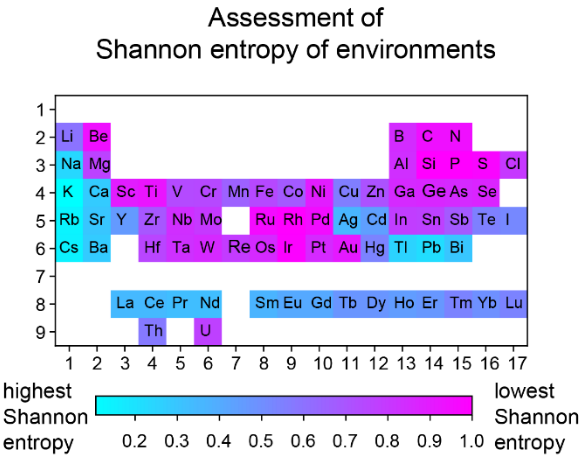

Figure S1. Shannon entropy for all assessed cations. A high Shannon entropy indicates that an element type shows many different coordination environments, a low Shannon entropy indicates that mostly one coordination environment dominates. For certain elements such as Si and P, only one coordination environment dominates.

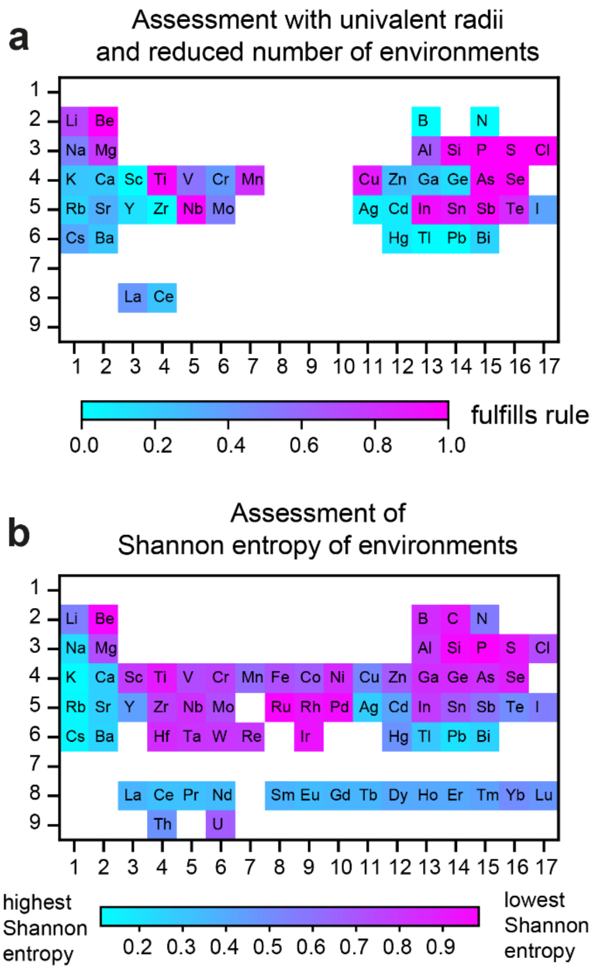

Figure S2. Assessment of the first rule. This time based on the experimental structures from the COD database.

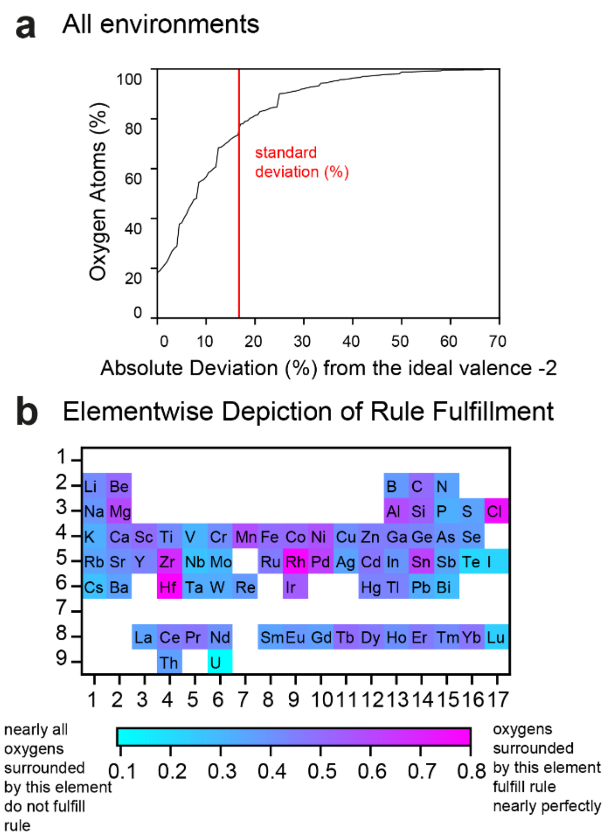

Figure S3. Assessment of the second rule. This time based on the experimental structures from the COD.

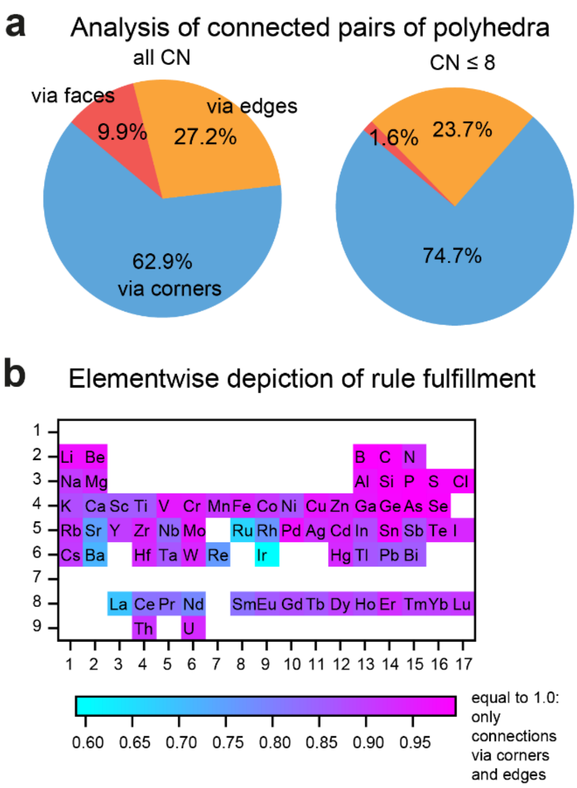

Figure S4. Assessment of the third rule based on the experimental structures from the COD.

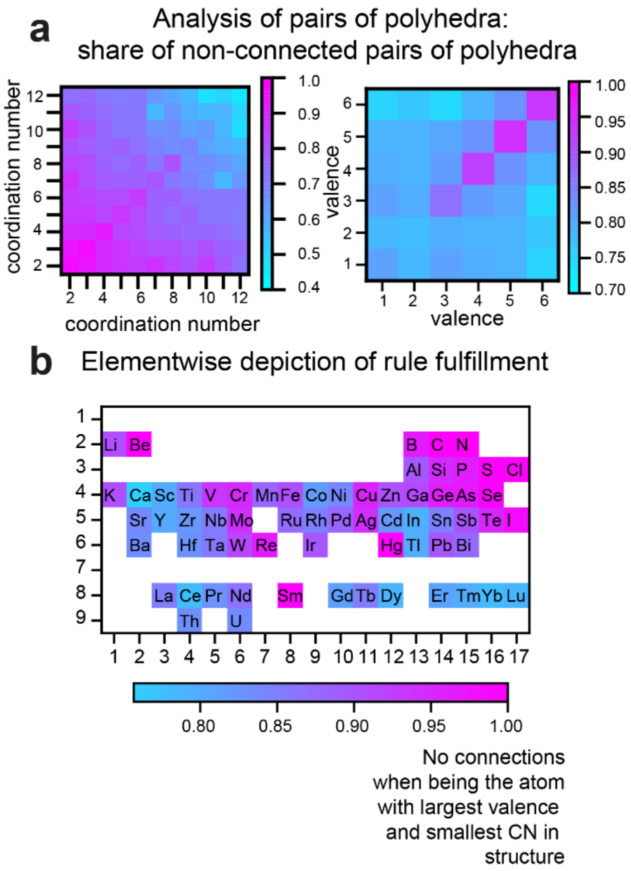

Figure S5. Assessment of the fourth rule based on the experimental structures from the COD.

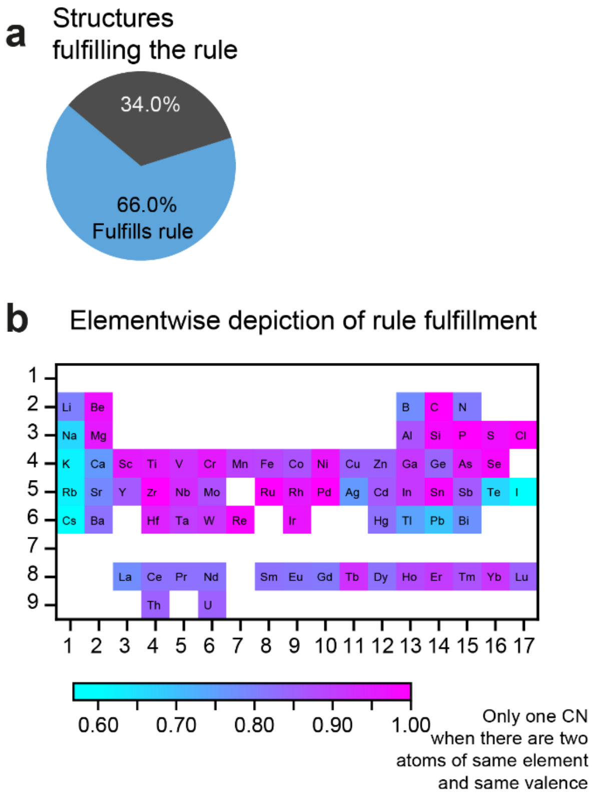

Figure S6. Assessment of the fifth rule based on the experimental structures from the COD.

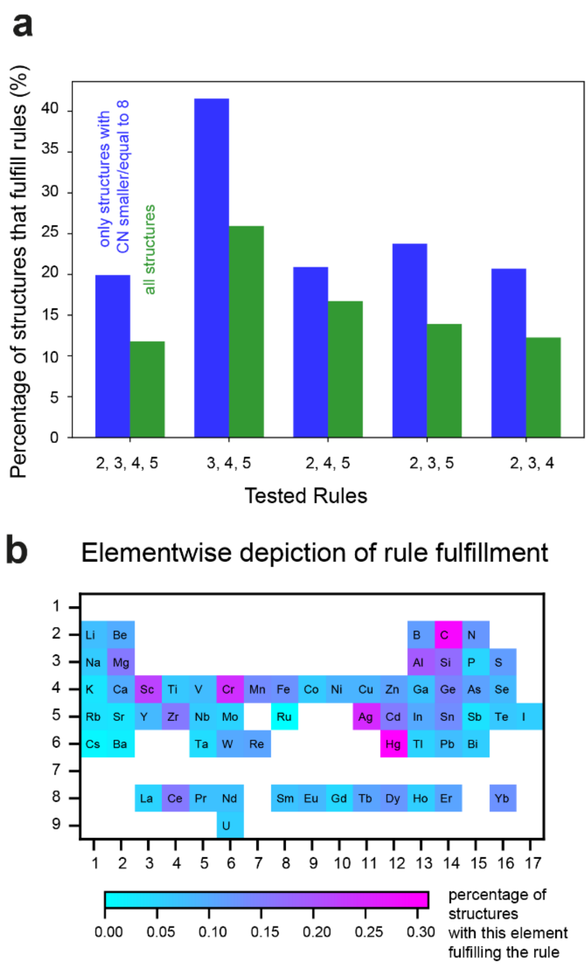

Figure S7. Assessment of the rules 2 to 5 based on the experimental structures from the COD.

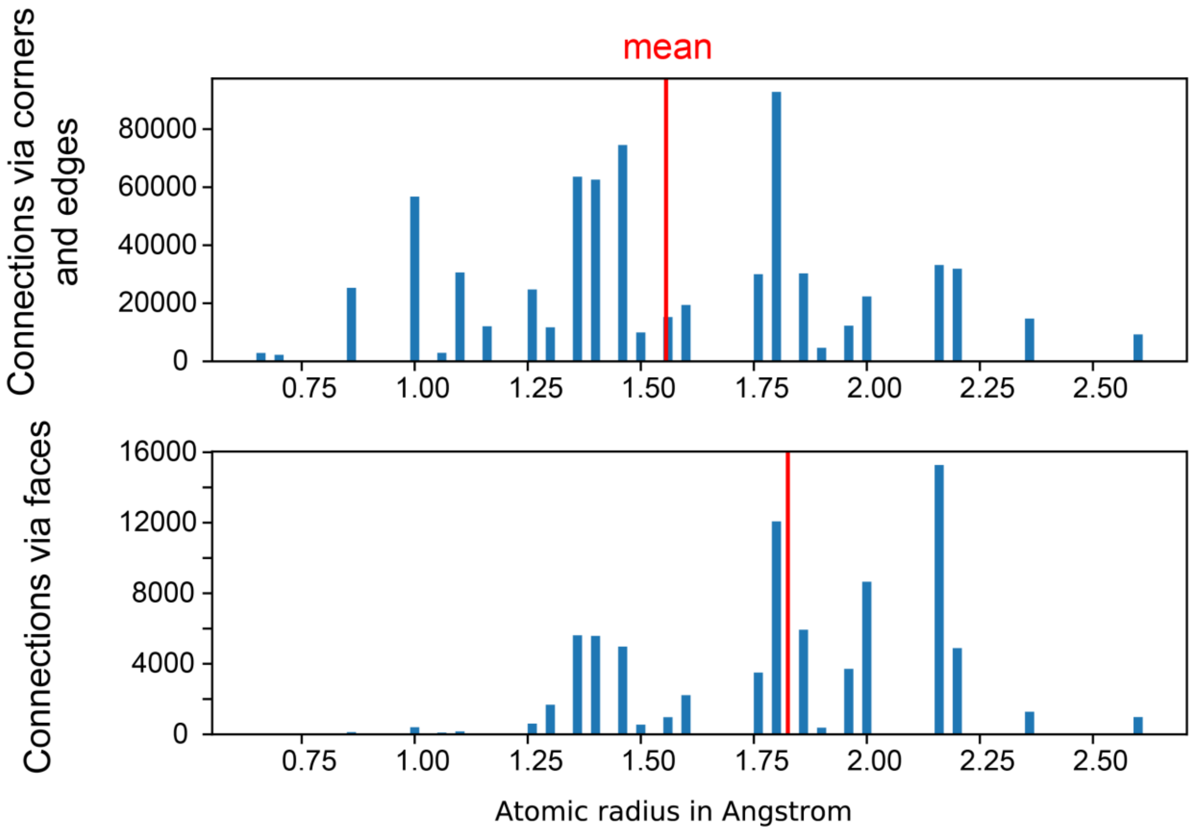

Figure S8. Connections via corners and edges, and connection via faces of the polyhedra pairs as a function of the atomic radius. One can see two different distributions.

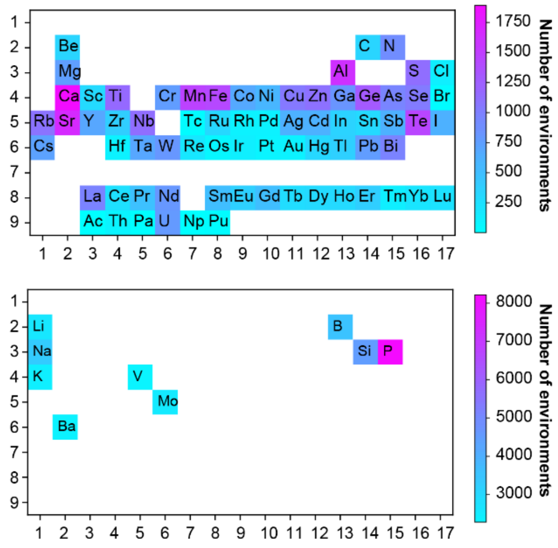

Figure S9. Number of environments considered in our dataset that is based on the Materials Project. top) Elements with number of environments between 0 and 2000. bottom) Elements with number of environments between 2000 and 8500.

References

[1] R. Allmann, R. Hinek, *Acta Cryst. A* **2007**, *63*, 412-417.

[2] R. T. Downs, M. Hall-Wallace, *Am. Mineral.* **2003**, *88*, 247-250.

[3] S. Grazulis, D. Chateigner, R. T. Downs, A. F. T. Yokochi, M. Quiros, L. Lutterotti, E. Manakova, J. Butkus, P. Moeck, A. Le Bail, *J. Appl. Crystallogr.* **2009**, *42*, 726-729.

[4] S. Gražulis, A. Daškevič, A. Merkys, D. Chateigner, L. Lutterotti, M. Quirós, N. R. Serebryanaya, P. Moeck, R. T. Downs, A. Le Bail, *Nucleic Acids Res.* **2012**, *40*, D420-D427.

[5] S. Gražulis, A. Merkys, A. Vaitkus, M. Okulič-Kazarinas, *J. Appl. Crystallogr.* **2015**, *48*, 85-91.

[6] A. Merkys, A. Vaitkus, J. Butkus, M. Okulič-Kazarinas, V. Kairys, S. Gražulis, *J. Appl. Cryst.* **2016**, *49*.

[7] S. P. Ong, W. D. Richards, A. Jain, G. Hautier, M. Kocher, S. Cholia, D. Gunter, V. L. Chevrier, K. A. Persson, G. Ceder, *Comp. Mater. Sci.* **2013**, *68*, 314.

[8] D. Waroquiers, X. Gonze, G.-M. Rignanese, C. Welker-Nieuwoudt, F. Rosowski, M. Göbel, S. Schenk, P. Degelmann, R. André, R. Glaum, G. Hautier, *Chem. Mat.* **2017**, *29*, 8346.

[9] K. Momma, F. Izumi, *J. Appl. Crystallogr.* **2011**, *44*, 1272–1276.

[10] L. Pauling, *The Nature of the Chemical Bond and the Structure of Molecules and Crystals: An Introduction to Modern Structural Chemistry*, Cornell University Press, **1960**.
